# Impact of a novel patient-specific, patient-matched Bezier parametric curve rod platform on proximal junction biomechanics in an *in silico* thoracolumbar instrumented fusion model

**DOI:** 10.1007/s43390-025-01146-4

**Published:** 2025-07-11

**Authors:** Franck Le Naveaux, Bahe Hachem, Sasha Vaziri, Varun Puvanesarajah, Saeed Sadrameli, David O. Okonkwo, Thomas J. Buell, Amit Jain, Hamid Hassanzadeh, Craig Forsthoefel, Reginald Fayssoux, Zachary J. Tempel, Alekos A. Theologis, Christopher S. Ahuja

**Affiliations:** 1 NumaLogics, Montreal, QC H2W 2R2 Canada; 2 Neurosurgery and Spine Specialists, Sarasota, FL USA; 3 Department of Orthopaedic Surgery, University of Rochester, New York, NY USA; 4 Orlando Neurosurgery, Kissimmee, FL USA; 5 Department of Neurological Surgery, University of Pittsburgh, Pittsburgh, PA USA; 6 Department of Orthopaedic Surgery, Johns Hopkins University, Baltimore, MD USA; 7 Illinois Bone and Joint Institute, Morton Grove, IL USA; 8 Desert Orthopaedic Center, Rancho Mirage, CA USA; 9 Mayfield Brain and Spine, Dent, OH USA; 10 Department of Orthopaedic Surgery, University of CA – San Francisco (UCSF), 500 Parnassus Ave, MUW 3Rd Floor, San Francisco, CA 94143 USA; 11 Department of Neurological Surgery, Northwestern University, Chicago, IL USA

**Keywords:** Adult spinal deformity, Patient-specific rods, Proximal junctional kyphosis, Biomechanics, Finite element analysis

## Abstract

**Purpose:**

To evaluate the biomechanical performance of a novel Bezier surface-smoothed transition rod, and to compare it to conventional and stepped rods, focusing on correction capability, spinal stabilization, instrumentation and spinal loading related to risk of proximal junctional kyphosis (PJK).

**Methods:**

A spine finite element model with patient-specific 3D spinal geometry (severe sagittal imbalance from thoracolumbar kyphosis) was used. Surgical instrumentation with five rod types was simulated: (1) constant 6.0 mm diameter, (2) stepped 6.0 mm–5.0 mm diameter, (3) Bezier 6.0 mm–5.5 mm–5.0 mm diameter, (4) constant 5.5 mm diameter, and (5) Bezier 5.5 mm–5.0 mm–4.75 mm diameter. Gravitational forces and flexion movements were simulated to compare load transfer between the spine and instrumentation.

**Results:**

All rod configurations achieved equivalent sagittal correction. Load distribution analysis showed that Bezier rods provided smoother load transitions and better offloading of proximal segments compared to constant diameter rods. The highest moment sustained by the segment adjacent to the instrumentation was observed with the constant 6 mm rod (9N.m), while the Bezier 5.5–5–4.75 mm rod showed the lowest moment (7.5Nm), indicating reduced stress of 16% on the upper adjacent vertebrae. Similarly, the Bezier rods were more effective in offloading pedicle screws up to 45% with respect to the stiffer rod construct, potentially reducing the risk of PJK.

**Conclusions:**

The simulation analysis demonstrates Bezier rods offer promising biomechanical benefits particularly in load distribution and stress reduction at adjacent levels of long thoracolumbar instrumentation. Future efforts will focus on clinical validation and optimization of patient-specific designs.

## Introduction

Spinal rods play a critical role in guiding and stabilizing the spine to promote spinal fusion. Currently, these rods are typically made from metal alloys such as titanium, cobalt–chromium, and stainless steel, or molybdenum–rhenium, each with varying Young’s modulus [1, 2]. The diameter of the rod is a key determinant of its bending rigidity, which increases significantly as the diameter increases. The choice of material and diameter together determine the flexural stiffness of the rod (Table 1). While stiff rods can help correct spinal deformities more effectively, excessive stiffness may contribute to complications such as stress shielding, implant loosening, and proximal junctional kyphosis (PJK) characterized by abnormal kyphosis at the upper adjacent segment of the instrumented spine [3, 4].

**Table 1:**
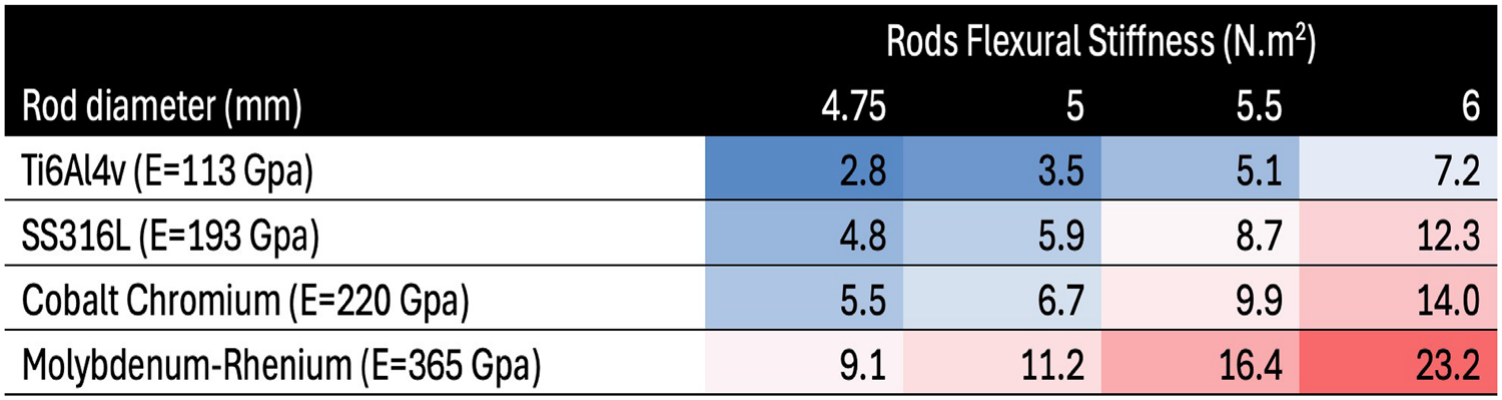
Theoretical rod stiffness values calculated using beam theory under flexion, accounting for the Young’s modulus (E) and the moment of inertia of a circular cross section

PJK is a well-recognized complication in spinal deformity surgery, occurring in 6%–62% of patients with potential added treatment costs of $55,547–$193,277 [3, 5–8]. Several factors have been linked to PJK, including bone density, pre-operative sagittal malalignment, fusion level, body mass index, smoking, and the stiffness of the construct. Among these, the stiffness of the construct, influenced by the choice of rods, is a risk factor that surgeons can control [4, 9–11].

Several studies have investigated various rod materials and diameters, yet there remains no consensus on the optimal rod design that balances correction capabilities with mitigating PJK risk [3, 12–14]. Most studies have focused on rods with consistent diameters, offering uniform stiffness along the spine. Stepped rods have been proposed to address these needs, but they pose challenges such as stress concentration at the transition, resulting in an increased risk of rod breakage and difficulties in screw placement [11, 14]. The introduction of Bezier surface-smoothed transition rods, which vary in stiffness based on local rod section diameter, aim to address these challenges. Bezier rods are patient specific and can be designed with greater stiffness where additional stiffness is needed (i.e., across 3-column osteotomies, multi-level posterior column osteotomies, lumbosacral junction) while maintaining flexibility near the upper instrumented vertebra so as to promote a biomechanical “soft landing” at the junction between the instrumented spine and native/non-instrumented spine.

The objective of this study is to evaluate the performance of the Bezier surface-smoothed transition rod compared to conventional and stepped rods in terms of correction capability, spinal stabilization and instrumentation, and spinal loading related to PJK risk. We hypothesize that this innovative rod design alleviates biomechanical stress at the instrumented–non-instrumented transition while maintaining robust correction capabilities. This study leverages *in silico* biomechanical analysis on patient-specific spinal finite element model, offering insights into load and stress distribution within the spine and instrumentation in a controlled and repeatable environment.

## Materials and methods

**Fig. 1:**
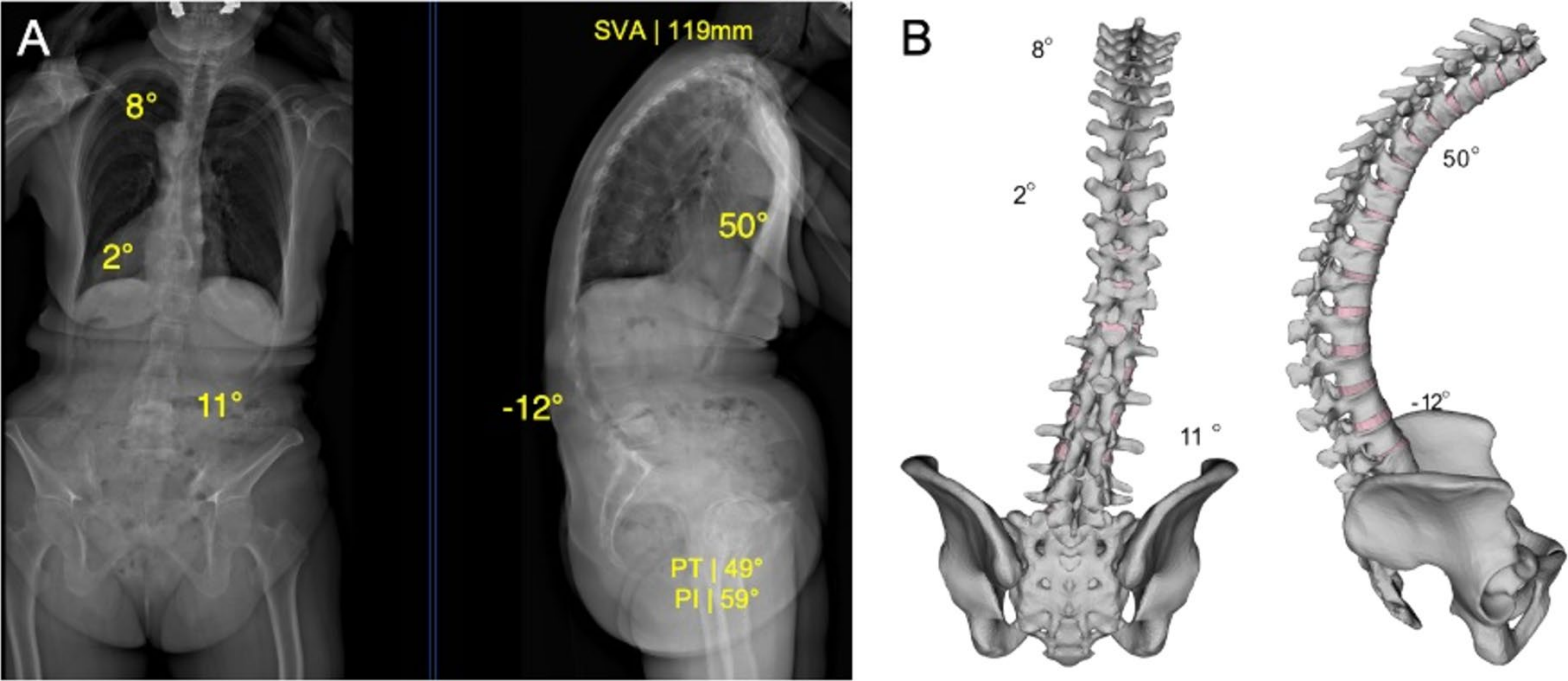
The finite element model is tailored to precisely replicate the 3D spinal geometry of individual patients, as determined from bi-planar radiographs. The mechanical properties of the model are customized at each vertebral level to accurately reflect the specific disc space dimensions and the corresponding range of motion of each segment

A spine finite element model (FEM) that has undergone multiple validation activities related to posterior spinal instrumentation to support previous research hypotheses was utilized [15–19]. Based on pre-operative standing posteroanterior and lateral calibrated radiographs, a patient-specific 3D spinal geometry of a 68-year-old female was reconstructed from T1 to the pelvis with a precision of 1.8 mm, followed by the constructions of an osteo-ligamentous finite element model. The patient’s morphology indicated a thoracolumbar kyphosis [type K Schwab ASD classification, pre-operative thoracic kyphosis T4–T12 (TK): 50°, lumbar lordosis L1–S1 (LL): − 12°; pelvic incidence (PI): 59°; pelvic tilt (PT): 49°] (Fig. 1) [20, 21].

**Fig. 2:**
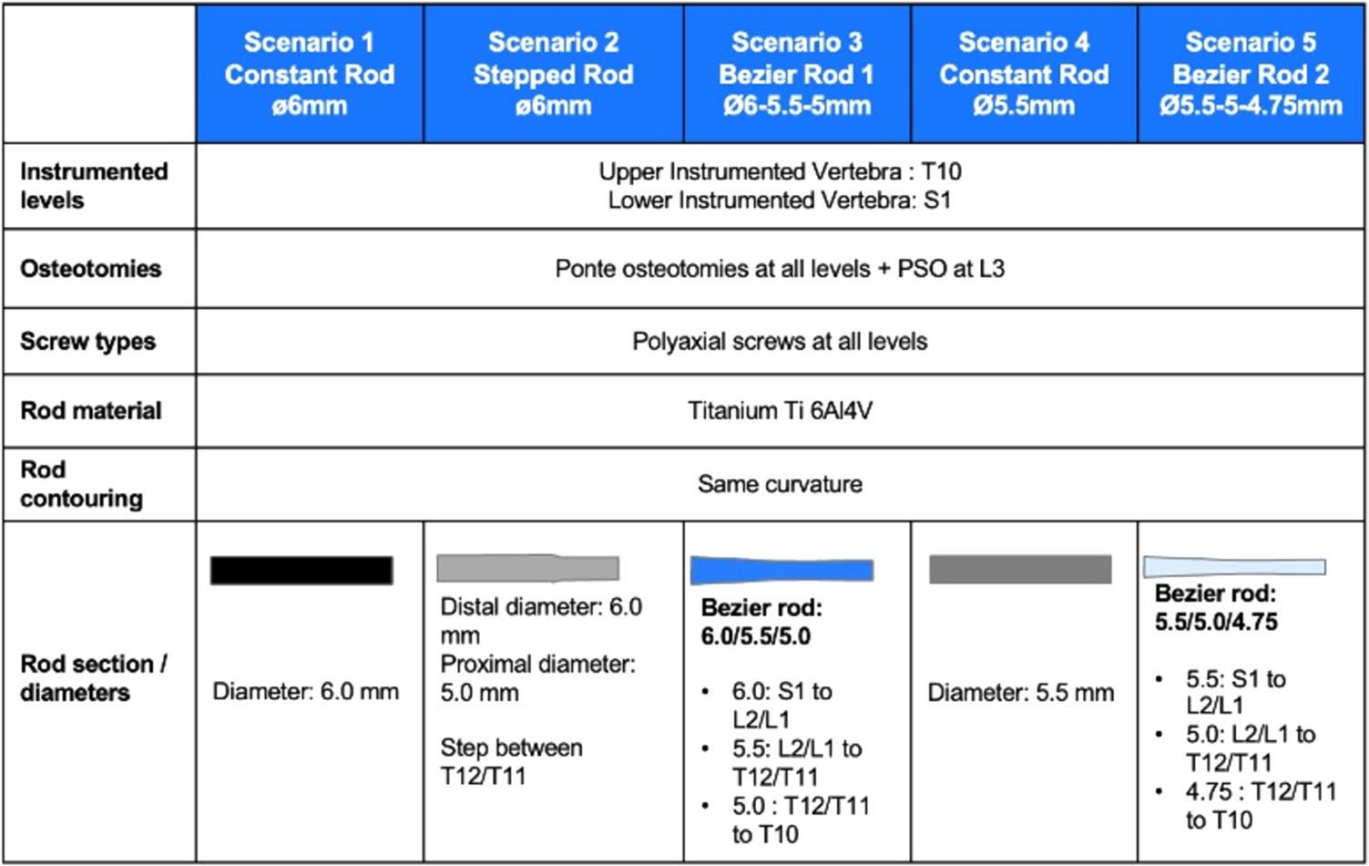
Five surgical strategies simulated. All instrumentation strategy parameters remained consistent except for the rod section profile along the instrumented levels

The pelvis and vertebrae were modeled as rigid bodies due to their minimal deformation during surgery. The FEM model did not simulate pelvic movement before and after surgical intervention. The non-linear behavior of each functional spinal unit, comprising two adjacent vertebrae and associated soft tissues, was defined with a 6 × 6 non-linear joint calibrated using data from cadaveric experimental studies [22–27]. Adjustment of the moment–rotation curves for each thoracic intervertebral unit incorporated a stiffening multiplier to reflect the biomechanical influence of the rib cage [28]. Spinal instrumented fusion surgery was simulated to assess the correction achieved by a posterior construct from T10 to pelvis using bilateral pedicle screw constructs and titanium rods with five different types of sections (Fig. 2).

Rod material was modeled with an elastoplastic multilinear stress–strain relationship to account for rod permanent deformation (E = 113 Gpa, yield stress = 950 Mpa, ultimate strength = 1180 Mpa at 10% elongation). The following five different scenarios based on the type of rod were evaluated:

1. Constant diameter rod of 6.0 mm.
2. Stepped rod with a distal diameter of 6.0 mm and a proximal diameter of 5.0 mm, with a step between T12 and T11.
3. Bezier rod with a diameter of 6.0 mm from S1 to L2/L1, 5.5 mm from L2/L1 to T12/T11, and 5.0 mm from T12/T11 to T10.
4. Constant diameter rod of 5.5 mm.
5. Bezier rod with a diameter of 5.5 mm from S1 to L2/L1, 5.0 mm from L2/L1 to T12/T11, and 4.75 mm from T12/T11 to T10.

**Fig. 3:**
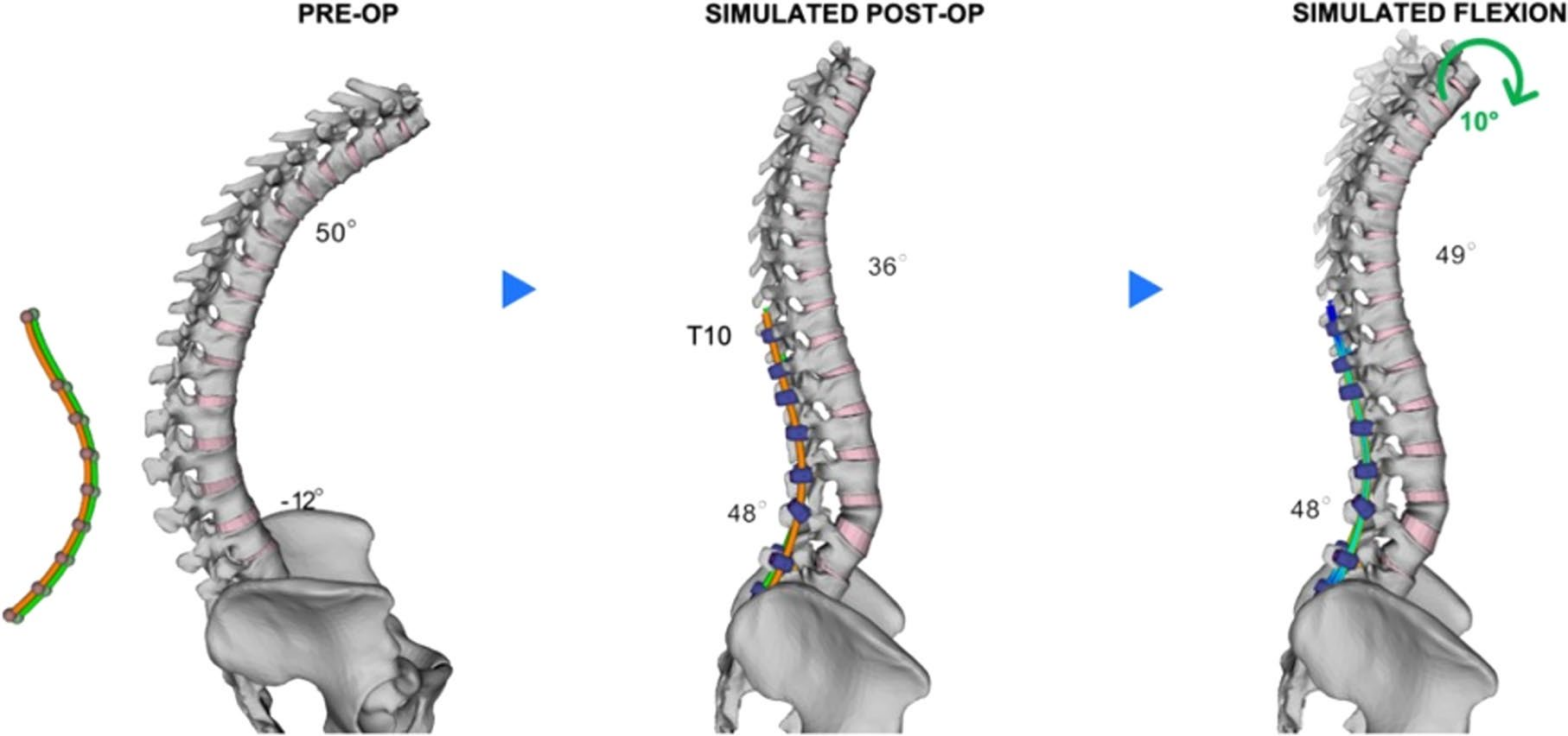
Simulation steps, from pre-operative spinal shape with rod contouring strategy similar for each simulated scenarios, simulated post-operative spinal shape in standing position, and simulated spinal shape under 10° flexion

The patient’s transition from a weight-bearing standing position to an intraoperative prone position was simulated [29–31]. Facetectomies were performed from T10 to S1, except at L3, where a pedicle subtraction osteotomy (PSO) was conducted to restore lumbar lordosis. Polyaxial screws were then positioned posteriorly in the vertebrae at the selected levels, followed by rod insertion and compression of the PSO at L3. Finally, the transition back to a standing and weight-bearing position was simulated (Fig. 3). Gravitational forces were applied in a follower-load manner to mimic physiological conditions [29]. Post-operative PT was defined based on PI with respect to Schwab and Le Huec equations for sagittal balance (*Schwab *et al*.* [32]: PT < 20° and *Le Huec *et al*.* [33]: PT = 0.44*PI – 11.4 = 15°). To further evaluate load distribution during functional movement, a similar flexion motion was simulated for each scenario by applying a forward rotation of 10 degrees at the T1 vertebral level (Fig. 3).

### Outcome metrics

The output metrics and related patient outcomes measured in this study focused on the following key areas.

1. Sagittal balance correction capability was assessed by comparing changes in lordosis and kyphosis between pre- and post-operative conditions.
2. Spinal stabilization capability was evaluated by examining the range of motion (ROM) of the instrumented spine under flexion.
3. PJK risk mitigation was analyzed by assessing the loading on spinal units and the ROM of the upper adjacent vertebrae. As such, their respective discontinuities under flexion and the loading on the upper instrumented vertebrae screws were compared.

Instrumentation failure risks were evaluated by measuring rod stresses and the forces sustained by pedicle screws. These metrics provide comprehensive insights into the biomechanical performance and potential clinical outcomes of different spinal rod configurations.

## Results

**Fig. 4:**
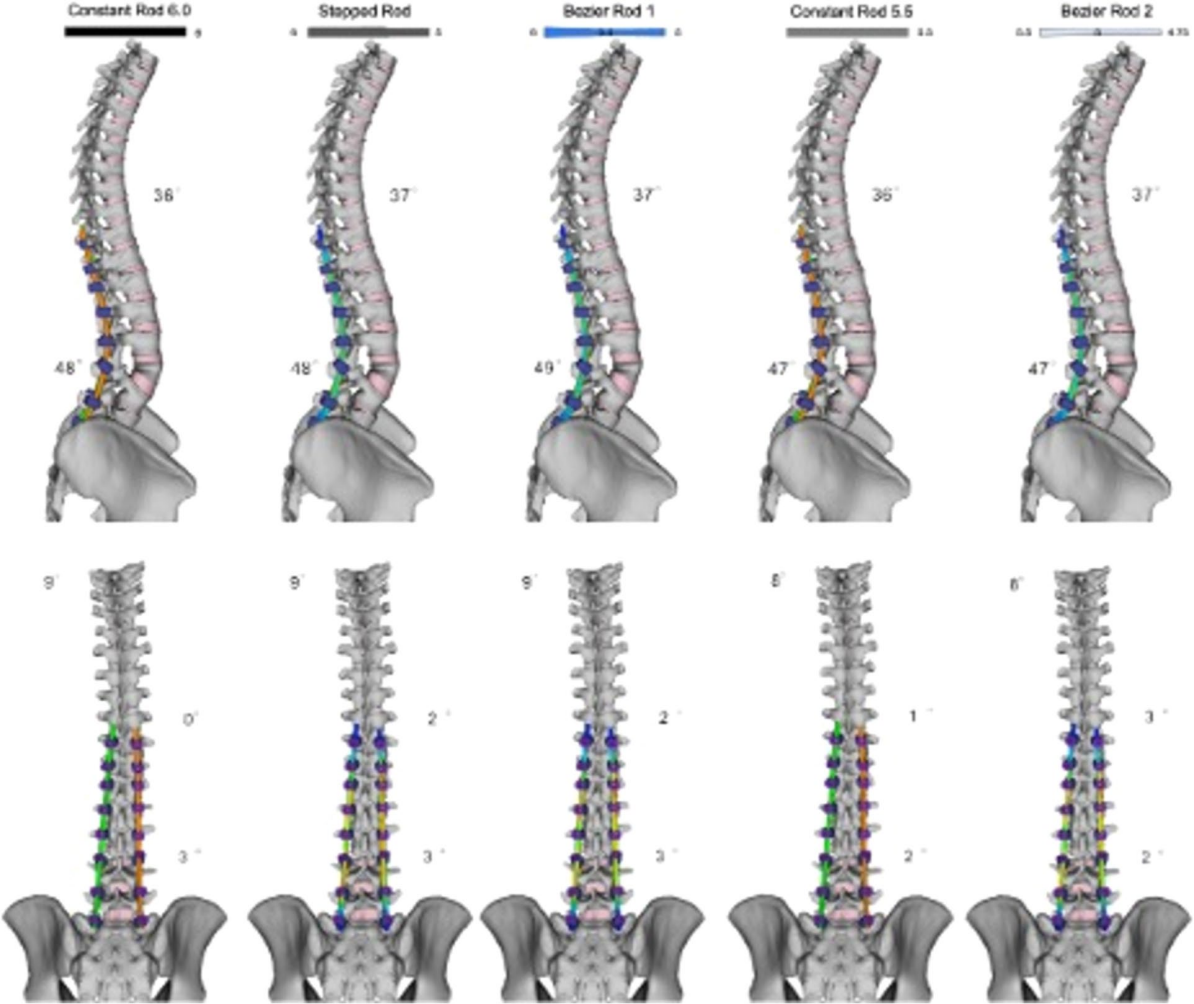
Post-operative standing spine in the sagittal plane following instrumentation with the five instrumentation scenarios

The simulated transition from standing to prone position resulted in a decrease in thoracic kyphosis (T4–T12) to 41° and an increase in lumbar lordosis (L1–L5) to -5°. The post-operative sagittal correction achieved with the five different strategies ranged from 36° to 37° for TK and from 47° to 49° for LL (Fig. 4). The pre-operative PI–LL mismatch of 72° (where PI = 60° and LL = − 12°) was reduced to an average of 12° across the different rod configurations. In the coronal plane, the lumbar Cobb angle ranged from 2° to 3°, with all strategies yielding similar outcomes in terms of correction.

**Fig. 5:**
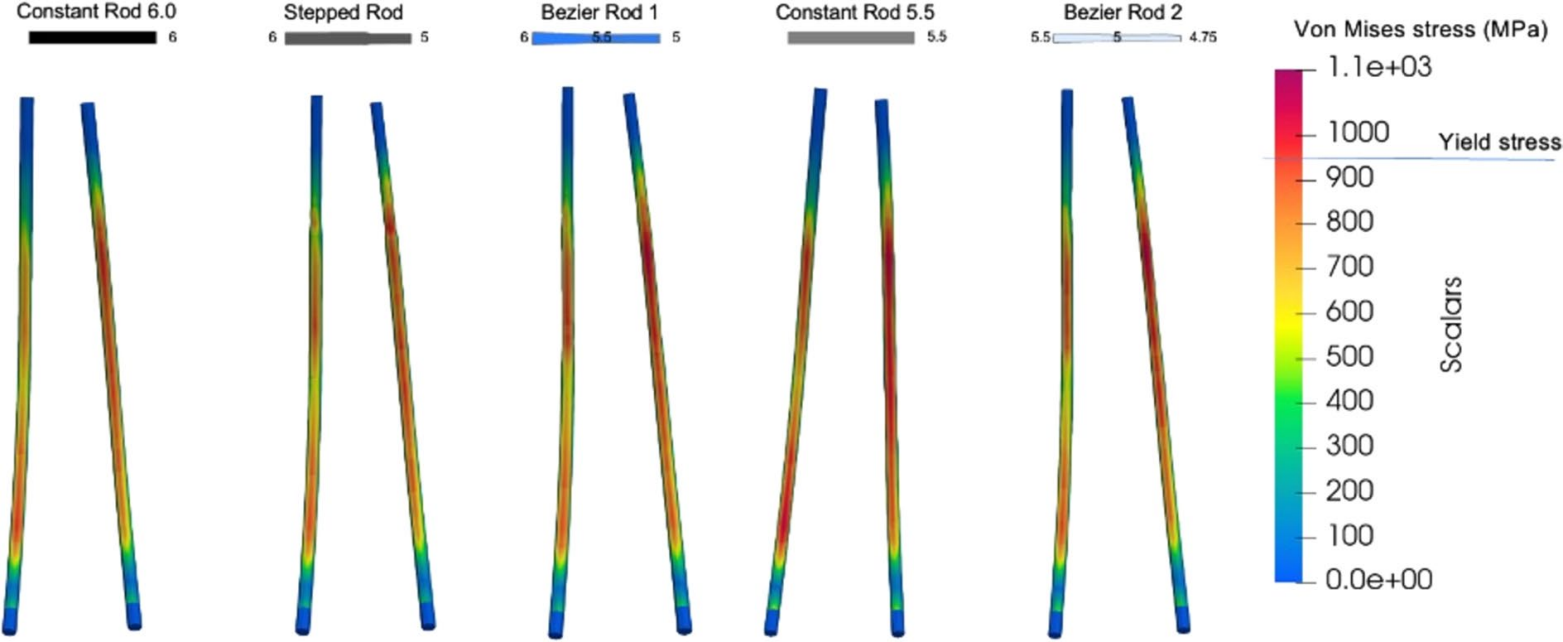
Stress distribution within the rods under standing position for the five simulated instrumentation scenarios

Rod deformation and stress distribution were also comparable across the simulated instrumentation scenarios, with stresses exceeding the yield stress of the material, indicating permanent deformation of the rods (Fig. 5). The stepped rod exhibited higher stress levels at the transition zone. Under flexion, the stabilization achieved by all constructs was equivalent. The highest range of motion in the instrumented segment was 0.36° at the upper instrumented segment, and a maximum of 0.10° at the levels below, demonstrating the ability of all constructs to stabilize the spine post-operatively.

In terms of loading, data for the intervertebral sagittal moment under flexion for the five instrumentation scenarios are reported in Table 2. The analysis focused on the moment sustained by the segment above the instrumentation where PJK might develop under 10° flexion. The highest moment sustained by the level adjacent to the vertebra was observed with the constant 6 mm rod (9.0 N.m), followed by the stepped 6 to 5 mm rod (8.7 N.m), the Bezier 6–5.5–5 mm rod (8.4 N.m), the constant 5 mm rod (8.2 N.m), and finally the Bezier 5.5–5-4.75 mm rod (7.5 N.m). In turn, proximal spinal loading was reduced by up to 16% with the Bezier 5.5–5-4.75 mm rod.

**Table 2:** Intervertebral sagittal moment (N.m) under flexion for the five instrumentation scenarios

	Constant 6.0 mm	Stepped 6.0–5.0 mm	Bezier ø6-5.5 mm-5.0 mm	Constant 5.5 mm	Bezier 5.5 mm-5 mm-4.75 mm
T1–T2	8.7	8.4	8.1	7.9	7.1
T2–T3	8.7	8.5	8.1	7.9	7.2
T3–T4	8.8	8.5	8.2	8.0	7.2
T4–T5	8.9	8.6	8.3	8.1	7.3
T5–T6	9.0	8.7	8.4	8.1	7.4
T6–T7	9.0	8.7	8.4	8.2	7.5
T7–T8	9.0	8.7	8.4	8.2	7.5
T8–T9	9.0	8.7	8.4	8.2	7.5
T9–T10 (UIV + 1)	9.0	8.7	8.4	8.2	7.5
T10–T11 (UIV)	4.5	6.5	6.6	5.5	5.5
T11–T12	4.3	3.4	2.5	4.4	1.6
T12–L1	5.4	5.8	4.0	4.3	3.0
L1–L2	2.5	2.1	2.3	2.1	1.9
L2–L3	NA	NA	NA	NA	NA
L3–L4	NA	NA	NA	NA	NA
L4–L5	2.6	2.6	2.1	3.0	2.4
L5–S1	10.9	10.7	10.7	10.3	9.5

*UIV* upper instrumented vertebra

Screw loads at each vertebral level are reported in Table 3. Different constructs resulted in varying screw loads by level, with the highest observed in the stepped 6 to 5 mm rod (477 N), followed by the the constant 6 mm rod (471 N), the Bezier 6–5.5–5 mm rod (453 N), the constant 5 mm rod (402 N), and finally the Bezier 5.5–5-4.75 mm rod (381 N). A similar trend was observed for screw forces at the UIV, with the highest load in the constant 6 mm rod (493 N), followed by the stepped 6 to 5 mm rod (388 N), the Bezier 6–5.5–5 mm rod (362 N), the constant 5 mm rod (331 N), and finally the Bezier 5.5–5–4.75 mm rod (270 N). Screw loading at the UIV was reduced by up to 45% with the Bezier 5.5–5–4.75 mm rod.

**Table 3:** Screw force magnitudes (newtons) under flexion for the five instrumentation scenarios

Vertebral level	Constant ø6		Stepped ø6-5		Bezier ø6-5.5–5		Constant ø5.5		Bezier ø5.5–5-4.75	
	Left screw forces (N)	Right screw forces (N)	Left screw forces (N)	Right screw forces (N)	Left screw forces (N)	Right screw forces (N)	Left screw forces (N)	Right screw forces (N)	Left screw forces (N)	Right screw forces (N)
T10	359	628	292	485	278	446	232	430	211	330
T11	145	233	152	167	131	151	231	214	162	190
T12	206	574	220	613	208	620	442	522	193	530
L1	183	180	423	251	193	232	109	145	180	243
L2	238	120	340	213	216	147	203	93	206	156
L3	NA	NA	NA	NA	NA	NA	NA	NA	NA	NA
L4	181	148	170	145	156	221	151	114	160	131
L5	1354	965	1283	930	1202	1084	1112	772	1061	763
S1	1111	918	1053	890	1004	964	918	752	867	719
Mean construct force (newtons)	471		477		453		402		381	

## Discussion

The current study underscores the importance of rod design in achieving optimal biomechanical outcomes and minimizing complications in spinal surgery. The ideal rod for spinal deformity surgery should have sufficient stiffness to allow surgeons to achieve goals of spinal realignment, without placing excessive forces on junctional transitions. In this study, we demonstrate *in silico* biomechanical results of a novel, patient-specific rod with continuous shaping which enables modulation of rod flexibility along the spinal segments. This design targets the selection of specific rod sections for segments requiring greater stiffness to correct deformities, while aiming at providing flexibility to smooth load transitions to adjacent segments.

While rod design is crucial, the extent of correction depends on patient factors (i.e., bone quality and spinal flexibility) and surgeon factors (i.e., soft tissue releases and bony osteotomies). This study’s results indicate that all rod configurations achieved similar correction in terms of lumbar lordosis and thoracic kyphosis. However, the load transfers between the instrumented segments and the adjacent, non-instrumented segments varied substantially based on the rod design. Specifically, the Bezier rods demonstrated a smoother load transition from the instrumented to the non-instrumented spine compared to constant diameter rods, leading to less stress on the proximal segments, which may result in lower PJK risk.

Moreover, PJK mitigating devices or approaches (e.g., tethers) aim to reduce excess spinal loading proximally and bring spinal loading closer to normal conditions, which was the tendency observed with the Bezier rods [34, 35]. Additionally, the Bezier rods were more effective in offloading the pedicle screws, especially the Bezier 5.5–5–4.75 scenario, which had the lowest implant loads. These variations in load distribution highlight the potential of Bezier rods to reduce stress levels and potentially further mitigate the risk of PJK, warranting further investigation into critical load thresholds and the physiological factors involved. Future research will focus on the additive effects of the Bezier rod constructs in combination with other PJK-prevention techniques [i.e., cement augmentation at the upper instrumented vertebra (UIV) and UIV + 1] to demonstrate possible superiority. Furthermore, further evaluation of tri- and quad-rod constructs are necessary to investigate differences between each of the rod constructs studied in this manuscript. This is especially important in the setting of extreme PI–LL mismatch and stiff sagittal deformity requiring PSO and multi-level SPOs.

The design of the rod is a critical factor in the performance of the instrumented spinal construct. While stepped rods were effective in offloading the proximal section compared to their constant section counterparts, they can, from a mechanical perspective, introduce stress concentrations at the transition points, increasing the risk of rod breakage under repeated loading conditions and complicating surgical maneuvers due to repeated screw engagement. In contrast, the Bezier rods, with their smooth transitions, provided a more gradual load shift between instrumented and non-instrumented spinal sections. This may be a benefit both for adult spinal deformity surgery, where proximal failure is a non-trivial event, but also for pediatric spinal deformity surgery, where both proximal and distal transitions can be tailored to reduce adjacent segment stresses to reduce PJK and distal junctional kyphosis failure modes. Further, the Bezier rods may provide a powerful solution for shorter, multi-level fusion constructs in degenerative lumbar fusion scenarios (such as L2 to pelvis fusion), to blunt adjacent segment stresses and avoid adjacent segment degeneration. Finally, Bezier rods share similarities with multi-rod constructs in that both strategies aim to optimize load distribution and enhance construct stability. While multi-rod constructs increase stiffness by adding supplementary rods, Bezier rods achieve a similar biomechanical effect by gradually modulating rod stiffness along the instrumented spine. While a potential limitation of Bezier rods is the constraint of maximum rod diameter (typically 6 mm), whereas multi-rod constructs can significantly increase stiffness by incorporating multiple rods spread across the spine, Bezier rods do not require additional implants beyond standard rod fixation systems, thus avoiding the added costs and surgical complexity associated with multi-rod constructs. While this practical advantage highlights the potential of Bezier rods as a cost-effective alternative for optimizing spinal fixation, while maintaining efficiency in surgical workflow, future research is required to validate these models and ascertain the magnitude of clinical benefit.

A major benefit to this study is that it leveraged a validated, *in silico* patient-specific model, providing a relevant clinical scenario while allowing for high control over the model input variables, enabling rigorous comparative analysis. This approach minimizes the experimental variability often encountered in cadaveric studies. However, while computational modeling provides valuable biomechanical insights, it is limited in its ability to predict post-operative sagittal balance changes, as these may be influenced by neuromuscular control, proprioception, and other compensatory mechanisms. Additionally, as our model extended up to T1, we are not able to comment on cervical sagittal alignment parameters and/or global sagittal alignment parameters that reference the cervical spine (i.e., cervical SVA). Another notable limitation is that only a single case was studied in which the PSO was performed at the L3 level. While we acknowledge that some correction objectives may favor achieving apical lordosis correction at L4 rather than L3, the simulated case scenario and PSO level were determined through a consensus among several surgeons in our group. While it may not be the preferred choice for all, an L3 PSO was deemed an appropriate surgical strategy for this case. To draw more definitive conclusions and understand the optimal balance between rod design and spinal stiffness, additional case simulations of varying complex three-dimensional deformity corrections with PSOs performed at different levels would be helpful and improve generalizability of the study. While exploring different surgical approaches may provide additional insights into the optimal construct, the selected surgical strategy does not affect the comparative nature of our study and its results, which focus on the biomechanical effects of different rod types, as the same strategy would be applied to all compared scenarios. An additional limitation of this study is that the model may be compromised by lack of inclusion of muscular forces, uncomplicated geometries of implants, and simplified interactions between different bone and implants. Additionally, the residual stresses from rod contouring and hardware tightening were not considered in this model. Ideal conditions were utilized for the screws and rods, which are rarely the case clinically. Noteworthy is that while this biomechanical analysis of this study was funded by a spine company who manufactures and sells the Bezier rods utilized in this analysis, the FE analysis was conducted by an independent laboratory *not* affiliated with the spine company and its results were in no way influenced by the spine company. Despite these limitations, this study may be considered a unique addition to the robust and growing literature on methods to mitigate proximal junctional failure in long thoracolumbar posterior instrumented fusions for spinal deformity.

## Conclusions

In this patient-specific finite element model, all rod configurations provided comparable correction capabilities for sagittal balance restoration, with Bezier rods showing a smoother load transition and reduced stress on proximal segments. This suggests that Bezier rods may offer superior performance in terms of reducing the risk of PJK compared to conventional rod designs. Future research should focus on validating these findings across a broader patient population to confirm the benefits of Bezier rods and better understand how specific rod sections should be tailored with respect to the different vertebral levels for optimal clinical outcomes.

## Data Availability

The data that support the findings of this study are available from the corresponding author, [AAT], upon reasonable request.
